# Clinico-radiological Observations in Meconium Aspiration Syndrome - Letter to The Editor

**DOI:** 10.31729/jnma.3541

**Published:** 2018-10-31

**Authors:** Jogender Kumar

**Affiliations:** 1 Newborn Unit, Nehru Hospital, PGIMER, Chandigarh, India

**Dear Editor**,

We read with interest the article “Clinico-radiological Observations in Meconium Aspiration Syndrome” in the recent issue of your esteemed journal and found it very useful.^1^ This article presents the morbidity profile of babies with Meconium Aspiration Syndrome (MAS). However, there are certain points we would like to highlight which might bring more clarity to this issue and will be useful to the readers.

1. The definition used by the authors includes the presence of Meconium-Stained Amniotic Fluid (MSAF) with either presence of meconium below vocal cord or having respiratory distress shortly after birth. It means babies born through MSAF even without respiratory distress were classified as having MAS. This definition is not correct for clinical practice. Classically Meconium Aspiration Syndrome is defined as respiratory distress in the newborn in the immediate neonatal period due to the presence of meconium in the trachea. This definition also covers babies with unexplained respiratory distress who are born through MSAF.^2^ All the studies included the presence of respiratory distress at birth as mandatory criteria for defining MAS.^3^ Inclusion of babies without distress doesn't justify their classification as MAS. Also, the author didn't mention any criteria for defining the severity of MAS.
2. One of the outcomes described is the incidence of birth asphyxia along with its severity in babies with MAS. However, the author didn't mention the definition used for defining birth asphyxia. From the description, it seems authors used Apgar scores for classifying as asphyxia. However, with our prior knowledge about the limitations of the Apgar score for predicting birth asphyxia, the detailed standard definition will be more useful.^4^ Also, authors planned to study immediate outcome and one of the important outcomes of interest in meconium aspiration is perinatal asphyxia leading to hypoxic-ischemic encephalopathy. So, it will be prudent here to describe how many babies in each group have hypoxic-ischemic encephalopathy.
3. This study was conducted using Neonatal Resuscitation Program (NRP) 2010 in which the management of birth depends upon the vigorous/non-vigorous status of the baby.^5^ The readership will be interested in knowing the number of babies who were vigorous and non-vigorous and their relationship with the thickness of the liquor.
4. The aim of the study was to understand and report the prevalence of meconium aspiration syndrome. For this, we need the total number of deliveries and number of babies born through MSAF in the hospital. Author described the proportion of MAS among NICU admission, not the prevalence of the MAS. Different NICU will have a different profile of the babies and the results cannot be generalized to other NICU's. However, if the author provides prevalence among all deliveries in their institute and proportion of MSAF who developed MAS later then it will have more epidemiological impact and will be more beneficial for the readership.
5. As written in methods, the authors gave oxygen and intravenous fluids to all MAS babies irrespective of the severity of the respiratory distress. Current literature didn't support this practice and advice starting of feeds as early as possible once distress is controlled.^6^ What was the rationale for withholding feeds in babies with minimal distress? In Table 2 authors mentioned that 23 babies didn't get oxygen. What was the reason for deviating from the standard management? If oxygen was not given, then how they were managed? Or they didn't have respiratory distress and got enrolled just because the meconium was noted below the vocal cords.
6. Author mentioned that in thick meconium group 52 (66.7%) babies had severe asphyxia. This data seems incorrect.
7. In Table 1, duration of stay in NICU (mean±SD) 4.0±2.6 days and 3.5±2.7 days in thin and thick MSL group, respectively is skewed data, so median instead of mean should be used.^7^
8. The whole story revolves around the thickness of meconium which itself is quite subjective and suffer interobserver variation. Various studies proposed different scoring/classification system to make it more objective.^8^ The evidence suggests that grading the severity of meconium staining by visual assessment has such poor accuracy and precision and it cannot provide a valid basis for assigning different care policies to different degrees of meconium staining.^9^ So, there is need of having further studies to have a uniform criteria for classifying meconium as thick or thin.

## Conflict of Interest

**None.**

## References

[1] Lama S, Mahato S, Chaudhary N, Agrawal N, Pathak S, Kurmi O, Bhatia B, Agarwal K. (2018). Clinico-radiological Observations in Meconium Aspiration Syndrome. J Nepal Med Assoc..

[2] Fanaroff AA. (2008). Meconium aspiration syndrome: historical aspects. J Perinatol..

[3] Natarajan CK, Sankar MJ, Jain K, Agarwal R, Paul VK. (2016). Surfactant therapy and antibiotics in neonates with meconium aspiration syndrome: a systematic review and meta-analysis. J Perinatol.

[4] (2014). Neonatal Encephalopathy and Neurologic Outcome. Pediatrics.

[5] Kattwinkel J, Perlman JM, Aziz K, Colby C, Fairchild K, Gallagher J (2010). Part 15: Neonatal Resuscitation: 2010 American Heart Association Guidelines for Cardiopulmonary Resuscitation and Emergency Cardiovascular Care. Circulation..

[6] Gidaganti S, A Faridi MM, Narang M, Batra P. (2018). Effect of Gastric Lavage on Meconium Aspiration Syndrome and Feed Intolerance in Vigorous Infants Born with Meconium Stained Amniotic Fluid- A Randomized Control Trial. Indian Pediatr..

[7] Manikandan S. (2011). Measures of central tendency: Median and mode. J Pharmacol Pharmacother..

[8] Amenu Sori D, Belete A. (2016). Meconium Stained Amniotic Fluid: Factors affecting Maternal and Perinatal Outcomes at Jimma University Specialized Teaching Hospital, South West Ethiopia. Gynecol Obstet..

[9] van Heijst ML, van Roosmalen G, Keirse MJ. (1995). Classifying meconium-stained liquor: is it feasible?.

